# Supplementary material for: The Oxford Needle Experience (ONE) scale: a UK-based and US-based online mixed-methods psychometric development and validation study of an instrument to assess needle fear, attitudes and expectations in the general public
Source: BMJ Open. 2023 Dec 13;13(12):e074466. doi: 10.1136/bmjopen-2023-074466 (PMC10729041; doi:10.1136/bmjopen-2023-074466)
Supplement: Supplementary data [file bmjopen-2023-074466supp001.pdf]

**Latent Variable**	**Item**	**Standardized Factor Loadings**	**Std. Err.**	**z-value**	**P-value**
Injection	1	1 (constrained)	-	-	-
Injection	2	0.897	0.043	20.77	<0.0001
Injection	3	0.878	0.044	19.92	<0.0001
Injection	4	1.149	0.042	27.43	<0.0001
Injection	5	0.673	0.052	12.99	<0.0001
Injection	6	0.997	0.042	23.78	<0.0001
Injection	7	0.859	0.052	16.50	<0.0001
Injection	8	1.180	0.043	27.52	<0.0001
Injection	9	0.658	0.048	13.68	<0.0001
Injection	10	0.955	0.048	19.73	<0.0001
Blood	11	1 (constrained)	-	-	-
Blood	12	0.928	0.063	14.80	<0.0001
Blood	13	1.008	0.057	17.58	<0.0001
History	14	1 (constrained)	-	-	-
History	15	1.135	0.044	25.89	<0.0001
History	16	0.837	0.051	16.36	<0.0001
Benefits	17	1 (constrained)	-	-	-
Benefits	18	1.860	0.243	7.66	<0.0001
Benefits	19	1.850	0.236	7.83	<0.0001

Supplemental Table. Standardized factor loadings of the ONE scale using structural equation modeling.
