# Supplementary material for: The Oxford Needle Experience (ONE) scale: a UK-based and US-based online mixed-methods psychometric development and validation study of an instrument to assess needle fear, attitudes and expectations in the general public
Source: BMJ Open. 2023 Dec 13;13(12):e074466. doi: 10.1136/bmjopen-2023-074466 (PMC10729041; doi:10.1136/bmjopen-2023-074466)
Supplement: Author's manuscript [file bmjopen-2023-074466.draft_revisions.pdf]

BMJ Open

BMJ Open is committed to open peer review. As part of this commitment we make the peer review history of every article we publish publicly available.

When an article is published we post the peer reviewers' comments and the authors' responses online. We also post the versions of the paper that were used during peer review. These are the versions that the peer review comments apply to.

The versions of the paper that follow are the versions that were submitted during the peer review process. They are not the versions of record or the final published versions. They should not be cited or distributed as the published version of this manuscript.

BMJ Open is an open access journal and the full, final, typeset and author-corrected version of record of the manuscript is available on our site with no access controls, subscription charges or pay-per-view fees (http://bmjopen.bmj.com).

If you have any questions on BMJ Open's open peer review process please email info.bmjopen@bmj.com

BMJ Open

Development and Validation of the Oxford Needle Experience (ONE) Scale

Journal:	*BMJ Open*
Manuscript ID	bmjopen-2023-074466
Article Type:	Original research
Date Submitted by the Author:	10-Apr-2023
Complete List of Authors:	Kantor, Jonathan; University of Oxford; University of Pennsylvania Perelman School of Medicine<br>Vanderslott, Samantha; University of Oxford<br>Morrison, Michael; University of Oxford<br>Pollard, Andrew; University of Oxford<br>Carlisle, Robert C; University of Oxford
Keywords:	Public health < INFECTIOUS DISEASES, Dialysis < NEPHROLOGY, Epidemiology < INFECTIOUS DISEASES

SCHOLARONE™
Manuscripts

I, the Submitting Author has the right to grant and does grant on behalf of all authors of the Work (as defined in the below author licence), an exclusive licence and/or a non-exclusive licence for contributions from authors who are: i) UK Crown employees; ii) where BMJ has agreed a CC-BY licence shall apply, and/or iii) in accordance with the terms applicable for US Federal Government officers or employees acting as part of their official duties; on a worldwide, perpetual, irrevocable, royalty-free basis to BMJ Publishing Group Ltd ("BMJ") its licensees and where the relevant Journal is co-owned by BMJ to the co-owners of the Journal, to publish the Work in this journal and any other BMJ products and to exploit all rights, as set out in our licence.

The Submitting Author accepts and understands that any supply made under these terms is made by BMJ to the Submitting Author unless you are acting as an employee on behalf of your employer or a postgraduate student of an affiliated institution which is paying any applicable article publishing charge ("APC") for Open Access articles. Where the Submitting Author wishes to make the Work available on an Open Access basis (and intends to pay the relevant APC), the terms of reuse of such Open Access shall be governed by a Creative Commons licence – details of these licences and which Creative Commons licence will apply to this Work are set out in our licence referred to above.

Other than as permitted in any relevant BMJ Author's Self Archiving Policies, I confirm this Work has not been accepted for publication elsewhere, is not being considered for publication elsewhere and does not duplicate material already published. I confirm all authors consent to publication of this Work and authorise the granting of this licence.

For peer review only

Development and Validation of the Oxford Needle Experience (ONE) Scale

Jonathan Kantor,[1,2,3*] Samantha Vanderslott,[1] Michael Morrison,[4] Andrew J Pollard,[1] Robert C Carlisle[2]

1. Oxford Vaccine Group, University of Oxford, Oxford, UK.
2. Biomedical Ultrasonics, Biotherapy, and Biopharmaceuticals Laboratory (BUBBL), Institute of Biomedical Engineering, Department of Engineering Science, University of Oxford, Oxford, UK.
3. Center for Clinical Epidemiology and Biostatistics, University of Pennsylvania Perelman School of Medicine, Philadelphia, PA, USA.
4. Centre for Health, Law, and Emerging Technologies (HeLEX), University of Oxford, Oxford, UK.

[*] Corresponding Author:

Jonathan Kantor, MD, MSc, MSCE
Oxford Vaccine Group
Biomedical Ultrasonics, Biotherapy, and Biopharmaceuticals Laboratory (BUBBL)
Institute of Biomedical Engineering
Department of Engineering Science
University of Oxford
Oxford OX3 9DU
United Kingdom
jonkantor@gmail.com
ORCID ID: 0000-0002-3256-3014

Author email addresses:

Jonathan Kantor: jonkantor@gmail.com ORCID ID: 0000-0002-3256-3014
Samantha Vanderslott: samantha.vanderslott@paediatrics.ox.ac.uk
Michael Morrison: michael.morrison@law.ox.ac.uk
Andrew J Pollard: andrew.pollard@paediatrics.ox.ac.uk
Robert C Carlisle: robert.carlisle@eng.ox.ac.uk

Funding: SV received funding from AHRC-IRC (HNR02360) and NIHR (HNR02910). RCC is grateful for the generous benefaction of Mr Donal Porteus which supported his contribution. The funding agencies and parties were not involved in study design, data collection, analysis, or presentation.
Data Statement: Data are available from the corresponding author
Conflicts of Interest: The authors have no conflicts of interest to declare.

Abstract

For peer review only

Objectives: To develop and validate the Oxford Needle Experience (ONE) scale, an instrument to assess needle fear, attitudes, and expectations in the general population.
Design: Cross-sectional validation study.
Setting: Internet-based with participants in the United Kingdom (UK) and United States (US).
Participants: UK and US representative samples stratified by age, sex, and ethnicity using the Prolific Academic platform.
Main outcome measures: Exploratory factor analysis with categorical variables and a polychoric correlation matrix followed by promax oblique rotation on the UK sample for the ONE scale. Confirmatory factor analysis (CFA) with a Satorra-Bentler scaled test statistic evaluating the root mean squared error of approximation (RMSEA), standardized root mean squared residual (SRMR), and comparative fit index (CFI) on the US sample. Reliability as internal consistency using McDonald's omega. Convergent and discriminant validity using logistic regression odds ratios of association (OR).
Results: The population included 1,000 respondents, 500 in the UK and 500 in the US. Minimum average partial correlation and a scree plot suggested 4 factors should be retained: injection hesitancy, blood-related hesitancy, recalled negative experiences, and perceived benefits, yielding a 19-question scale. On CFA, the RMSEA was 0.070 (90% confidence interval, 0.064, 0.077), SRMR 0.053, and CFI 0.925. McDonald's omega was 0.92 and 0.93 in the UK and US samples, respectively. Convergent validity with a single-question COVID-19 vaccination status question demonstrated a strong association, OR (95% CI) 0.97 (0.96, 0.98), p<0.0001 in the US sample. Discriminant validity with a question regarding the importance of controlling what enters the body confirmed the ONE score does not predict this unrelated outcome, OR 1.00 (0.99, 1.01), p=0.996 in the US sample.
Conclusions: The ONE scale is a valid and reliable multidimensional scale that may be useful in predicting vaccine hesitancy, designing public health interventions to improve vaccine uptake, and exploring alternatives to needles for medical procedures.

Strengths and limitations of this study:

- Using an iterative qualitative methodological approach to domain development followed by representative samples for analysis permits the inclusion of a broad and diverse stakeholder group in the development of the Oxford Needle Experience (ONE) scale.
- The multidimensional nature of the ONE scale means that public health researchers may be better positioned to understand the various contributors to needle hesitancy and further investigate opportunities for engagement to mitigate downstream vaccine hesitancy.
- The feasibility of the scale coupled with its multidimensionality and generalisability between the two populations studied may broaden opportunities for ONE scale adoption.
- In the US population, each 1-point increase in ONE score is associated with a 3% increase in the odds of being unvaccinated, translating into a 49.5% increase for each standard deviation increase in ONE score—highlighting the scale's excellent convergent validity and suggesting opportunities for immediate deployment in vaccine research.

For peer review only

For peer review only - http://bmjopen.bmj.com/site/about/guidelines.xhtml

- Future cross-cultural and international validation studies, as well as test-retest and cut-off studies, may be helpful to establish the ONE scale's utility in a broad range of populations.

Keywords: needles; needle phobia; anxiety; blood-injection-injury; fear; vaccine hesitancy; vaccines; public health; validation study; behaviour rating scale; venepuncture; phlebotomy; dialysis; local anaesthetic

Word Count: 2,982
References: 43
Tables: 5

For peer review only

Introduction

Vaccine hesitancy was identified by the World Health Organization as one of the top ten threats to public health even before the onset of the COVID-19 pandemic, and the potential impact of this phenomenon on global health has only increased in recent years.[1] While vaccine hesitancy is a complex and multifactorial phenomenon, previous work has suggested that injection fear may represent its only modifiable risk factor.[2,3] Given the importance of behavioural considerations in modulating vaccine acceptance, changes in methods of vaccine delivery may decrease vaccine hesitancy, leading to significant global health benefits.[4] With upwards of 10 billion vaccinations given by needle each year, better understanding the role of needles as potential contributors to vaccine hesitancy is of urgent public health importance, particularly as alternative approaches for vaccine delivery are undergoing development.[5-7]

Despite the central importance of understanding public attitudes to needle use for vaccination and other medical procedures, little is known regarding the myriad contributors to this phenomenon, and most previous scales designed to study needle attitudes, experiences, and fear were developed without fundamental qualitative research, in the absence of a multidimensional approach—often focusing exclusively on needle fear, anxiety, or even phobia—or in populations with suboptimal generalisability.[8-15]

Validated multidimensional scales are a useful tool to study behavioural phenomena, and may provide insight with direct global health implications.[16] Our goal, therefore, was to develop a feasible multidimensional scale that would capture the key domains contributing to concern regarding needle use in the context of vaccination or medical procedures. By better elucidating the underlying domains that may modulate vaccine hesitancy, and medical procedure hesitancy more broadly, these data may ultimately be used to better inform the development of evidence-based vaccination strategies to mitigate some of the perceived negative aspects of vaccine delivery. Moreover, a better understanding of the contribution of needles to vaccine hesitancy may also encourage the development of alternative vaccine-delivery technologies, ultimately resulting in more tailored and publicly acceptable approaches to vaccination and medical procedures in the future.

Methods

Domain and Item Generation

Domain generation to establish the domains of interest for the ONE scale was accomplished through an iterative deductive and inductive approach: after a literature review was performed, online focus groups were used to qualitatively develop a set of possible themes, and ultimately domains, that putatively contribute to needle attitudes, experiences, and expectations. A group of experts from the fields of medicine, public health, social science, and engineering science was consulted regarding domain development and inclusion, consistent with established scale development methodology.[16-18]

Participant item responses were recorded on a 5-point Likert scale ranging from strongly agree to strongly disagree. Items were reverse scored, as appropriate, to yield a scale where higher values represent greater levels of concern regarding needle use. This work was approved by the University of Oxford Medical Sciences Interdivisional Research Ethics Committee (approval reference R81585/ RE001). All subjects provided informed consent for participation and were permitted to withdraw from their anonymous surveys at any time. Qualitative

For peer review only - http://bmjopen.bmj.com/site/about/guidelines.xhtml

analyses were performed using NVivo release 1.4 (QSR International, Burlington, Massachusetts, USA) and statistical analyses were performed using Stata for Mac version 16 (Stata Corporation, College Station, Texas, USA).

Data Collection

Two separate representative samples were used in the development of the ONE. A sample of 500 respondents in the United Kingdom (UK) was recruited through Prolific Academic (Oxford, UK), an academic survey panel site, and a draft scale was presented using the Qualtrics platform (Qualtrics Corp, Provo, Utah, USA). All respondents were rewarded with a small payment (<£2). This sample was used to develop the scale and perform exploratory factor analysis (EFA). A second demographically representative sample of respondents from the United States (US) was then solicited in a similar fashion using the same electronic survey recruitment tools. This sample was used for confirmatory factor analysis and reliability and validity testing. Results from the US and UK were evaluated separately to establish the robustness of the scale. Demographic information was self-reported by respondents and was linked with responses through an anonymous 25-digit alphanumeric key. Baseline information included age, self-reported sex, self-reported race and ethnicity, employment status, education history, household income, and vaccination status against COVID-19.

Domain and Item Reduction and Exploratory Factor Analysis

The Kaiser-Meyer-Olkin procedure was conducted to ensure sampling adequacy.[19] To establish the number of domains to retain for the ONE scale, we calculated the minimum average partial correlation for the number of principal components, developed a scree plot, and performed parallel analysis for principal components.[18,20,21] Item reduction was approached by assessing domain relevance, examining polychoric (item-item) and polyserial (item-test) correlations, culling redundant items, and removing those with low communality or low factor loadings.[22] EFA with categorical variables was performed by using polychoric correlations to generate a factor matrix using iterated principal factor analysis followed by promax oblique factor rotation.[23,24]

Confirmatory Factor Analysis

The robustness of the findings was tested by performing confirmatory maximum likelihood factor analysis using structural equation modelling on the US sample with the model developed in the UK sample with a Satorra-Bentler scaled test statistic. Multiple goodness of fit indices were analysed, including the root mean squared error of approximation (RMSEA), the standardized root mean squared residual (SRMR), and the comparative fit index (CFI) to assess model fit. Values of RMSEA≤0.08, SRMR≤0.08, and CFI≥0.90 suggest acceptable fit.[25,26]

Reliability and Validity

Reliability as internal consistency was assessed by examining McDonald's omega, a non-parametric alternative to Cronbach's alpha, for each subscale domain and for the overall scale, in both the UK and US samples.[27]

Convergent validity was assessed by examining the association between the ONE scale score and vaccination status against COVID-19. Logistic regression odds ratios of association were calculated with vaccination status (defined as having received a minimum of one dose of any vaccine for COVID-19) coded as a dichotomous variable and the ONE scale treated as a continuous predictor variable. Discriminant validity was assessed by examining the logistic regression odds ratios of association between the ONE score and agreement with a non-specific

For peer review only - http://bmjopen.bmj.com/site/about/guidelines.xhtml

statement ('it is important to control what enters one's body') in the UK and US samples. A p-value of 0.05 was considered significant.

Patient and Public Involvement

This study was conducted to develop a valid scale to assess public attitudes to needle use for medical procedures such as vaccination. Patient and public involvement was not actively solicited for the initial phase of study design, and patients and members of the public were primarily participating as survey respondents. However, patients and members of the public taking part in online focus groups on needle hesitancy were able to provide feedback on the aims of the project as well as the suitability of the wording of specific scale items. The aim of this research is to develop a valid and reliable scale that reflects the priorities and preferences of the public. Results will be disseminated and may potentially be used in future studies to better assess patient and public preferences regarding the use of needles in vaccination and in medical procedure more broadly.

Results

Respondent Characteristics

In the UK sample, of the 506 respondents who started the survey, 500 subjects returned completed surveys, yielding a 98.8% completion rate in the survey panel. The median (interquartile range [IQR]) age was 45 (32, 59), and respondents ranged in age from 18 to 90. 50.4% of respondents identified as female, 48.2% as male, and 1% as non-binary. The median (IQR) self-reported household income was £39,000 (25,000, 58,000). A total of 47.6% of respondents were currently working full-time and 14.2% identified as non-white.

In the US sample, of the 509 respondents who started the survey, 500 subjects returned completed surveys, yielding a 98.2% completion rate in the survey panel. The median (IQR) age was 45 (31, 60), and respondents ranged in age from 18 to 84. 49.6% of respondents identified as female, 47.6% as male, and 1.6% as non-binary. The median (IQR) self-reported household income was $53,500 (31,000, 89,999). A total of 45.5% of respondents were currently working full-time and 27.8% identified as non-white.

Domain and Item Reduction and Exploratory Factor Analysis

A total of 16 themes were identified as potentially contributing to public attitudes to needles after literature review and qualitative analysis of online focus group data. A total of 107 items for potential scale inclusion were then developed for testing; the intentionally large domain and item pool was designed to pilot similar iterations of items in a focus group format. A total of 88 items were ultimately deleted due to low domain relevance, low polychoric or polyserial correlations, unifactorial outcomes, redundancy, or low communality.

Factor loadings from the 19 retained items after EFA for categorical variables with promax oblique rotation are presented in Table 1. The Kaiser-Meyer-Olkin measure of sampling adequacy was excellent, with a value of 0.92 for the overall model and with values ranging from 0.68 to 0.97 for each of the individual items. This 4-factor solution reflected a simple structure with appropriate loading and was supported by the results of the minimum average partial correlation analysis, the scree plot, and the Eigenvalue > 1 approaches to factor extraction.[28,29] Parallel analysis suggested retaining 3 factors, though this result was considered borderline given the adjusted Eigenvalue of 0.97 for the fourth factor, just below the cut-off of 1.[20]

For peer review only - http://bmjopen.bmj.com/site/about/guidelines.xhtml

Factors correlated clinically with the domains of 1) injection hesitancy (including fear and anxiety related to injection); 2) blood-related hesitancy; 3) recalled negative experiences; and 4) perceived benefits of needles.

Factor intercorrelations are included in Table 2, and the final ONE scale and scoring key are presented in Table 3.

Confirmatory Factor Analysis

Confirmatory factor analysis was performed on the US sample using the model developed in the UK sample. The maximum likelihood factor analysis model created using structural equation modelling demonstrated acceptable goodness of fit characteristics, with RMSEA=0.070 (90% confidence interval 0.064, 0.077), SRMR=0.053, and CFI=0.925 using the 4-factor model with a total of 19 items.

The median (interquartile range [IQR]) total ONE score was 37 (29-48) in the UK population and 39 (29-54) in the US population (p=0.054). Further descriptive statistics for each subscale are included in Table 4.

Reliability and Validity

Reliability as internal consistency was excellent: McDonald's omega for the overall ONE scale was 0.92 in the UK sample and 0.93 in the US sample. Omega values for each subscale are included in Table 5.

Convergent validity with the single-question vaccination status question demonstrated a significant association between vaccination status and ONE score, with a logistic regression odds ratio of association (95% CI) of 0.98 (0.96, 1.0), p=0.014 for the UK sample and 0.97 (0.96, 0.98), p<0.0001 for the US population. Given that the standard deviation of the ONE score in the UK and US samples is 15.2 and 16.5, respectively, this translates into a decrease in the odds of being vaccinated of 30.4% in the UK and 49.5% in the US for each single standard deviation increase in ONE score.

Discriminant validity with a question regarding the importance of controlling what enters the body confirmed that the ONE score does not predict this unrelated outcome, with logistic regression odds ratios of association of 1.01 (1.00, 1.02), p=0.162 in the UK population and 1.00 (0.99, 1.01), p=0.996 in the US sample.

Discussion

We have shown here that the ONE scale is a valid and reliable multidimensional instrument to assess public attitudes to needle use in the context of medical procedures such as vaccination. Given that injection fears may be the primary modifiable risk factor for vaccine refusal,[3] understanding the contributors to such responses is of vital public health importance. Moreover, needle concerns are not limited to those undergoing vaccination, and have been implicated in hesitancy regarding a range of procedures from venepuncture[30] to local anaesthetic injection in dermatologic surgery,[31] blood donation,[32] dental procedures,[33] and dialysis.[34] Therefore, a better appreciation of the myriad contributors to the needle exposure experience may be broadly valuable.

In evaluating descriptive statistics for each of the subscales, only the median scores for a recalled history of negative experiences differed significantly between the UK and US populations, with an absolute 33% increase reported in the US group (p<0.0001). As such recall has been previously implicated as a potential driver of needle fear—where a feedback loop of

For peer review only - http://bmjopen.bmj.com/site/about/guidelines.xhtml

recalled negative experiences leads to avoidance, furthering fear—this may have important implications for needle acceptance.[5] Moreover, the magnitude of the association between ONE score and COVID-19 vaccination status—the odds of being unvaccinated increase by almost 50% for each standard deviation increase in ONE score in the US population—not only highlights the convergent validity of the scale but also underscores its utility and potential value in designing public health interventions and the potential role of needle hesitancy as a contributor to vaccine hesitancy.[3]

Several prior instruments have been developed to assess needle attitudes. The 18-item Injection Phobia Scale-Anxiety (IPS-Anx) was developed in 1992 on a sample of 40 subjects with known needle phobia and examines fear and anxiety exclusively.[15] Two decades after its initial development, the factorial structure of the scale was assessed, suggesting a two-factor structure, encompassing contact (direct injection) and distal (watching or discussing injection) fears.[35] The 50-item Medical Fear Survey examines an array of fears relating to medical procedures, including injections as well as blood, physical examination, and mutilation;[36] an abbreviated version of this survey has also been developed.[10] The blood/injection fear scale was developed on a Turkish population of patients attending a hospital for examination or blood donation and is a 20-question scale that includes two domains, injection and blood.[12] The Blood-Injection Symptom Scale assesses domains of faintness, anxiety, and tension, and relied on a population of Australian university students for its development.[8] The 40-item Multidimensional Blood/ Injury Phobia Inventory (MBPI) was developed on a US university student population to assess phobias, and is aimed at assessing pathological reactions, rather than understanding the range of experiences of the general population exposed to needles.[9,11] Blood-injection-injury (BII) was also assessed as part of a 10-item subscale of the Specific Phobia Questionnaire, a lengthy scale addressing phobias ranging from fear of animals to the natural environment; this scale was developed on a cohort of patients with known anxiety disorders and a group of Canadian university students.[13] Finally, the multidimensional fear of injection scale was developed on 419 Japanese university students to evaluate fear of injections exclusively, encompassing domains of direct fear, indirect fear, physiological response, and avoidance.[14]

Unlike these scales, we used a qualitative methodology to ensure that our domain- and item-generation would include the diversity of priorities of a wide range of respondents to the experience of undergoing injection with a needle—a methodology designed to capture the broadest possible themes that may not be obvious to investigators a priori.[17,37-39] This is important, as prior studies have suggested a disconnect between clinicians' perception of patient or public priorities and their actual priorities.[40,41] The ONE scale is also the only instrument to include domains that assess recalled negative experiences as well as positive beliefs regarding the utility of needles, which may add significantly to researchers' understanding of patient and public priorities.

Additional strengths of our validation study include the iterative approach and the use of representative populations in our exploratory and confirmatory factor analysis; many prior studies relied on less diverse populations of young, well-educated students—or, conversely, on those being treated for anxiety disorders—and therefore the generalisability of their scales to the wider population is unknown. Furthermore, some prior scales aimed to evaluate pathological responses to needle injection—needle phobia. While such work is highly valuable,

For peer review only - http://bmjopen.bmj.com/site/about/guidelines.xhtml

our goal was to develop a scale that could be broadly adopted for designing public health interventions and alternative vaccine and medical therapeutic delivery technologies for the public.

We also relied on non-parametric statistical analyses, including a polychoric-matrix based EFA, to provide conservative estimates given concerns regarding the assumed normality of Likert-scale data.[42,43] This represents a departure from earlier studies that acknowledged the skewness inherent in needle fear assessments—the majority of individuals have only mild symptoms while a small proportion have extreme reactions—but used parametric statistics that assume normality in data analysis.[11]

Our work has several important limitations. First, we relied on a survey panel design to allow for a demographically representative population from both the UK and US, but there may be important differences between the general population and the subset willing to volunteer to contribute to survey panels, and response rates cannot be calculated with this approach. Moreover, all research that relies on self-report introduces the risk that bias, and particularly response bias and social desirability bias, may affect stated preferences. Nevertheless, the robustness of our findings, with total and subscale scores largely similar across both the UK and US populations, supports the generalisability of the scale. Second, one domain—injection hesitancy—accounts for 10 of the 19 items in the scale, while the remaining domains each include 3 items. The unequal number of items across domains, however, was purposely designed to capture the presence of a primary domain of interest—injection hesitancy—while also acknowledging and evaluating the contribution of other domains. Indeed, some have suggested that a unitary construct underlies responses to needle injection,[11] and it was our goal to expand on this central domain rather than attempt to over-extract factors that otherwise correlate closely. Third, additional validation testing, as well as test-retest reliability and cross-cultural validation studies, should be considered in the future to better understand the limits of the ONE scale's generalisability.

The ONE scale addresses four separate domains that contribute to the public's experience with needles: injection hesitancy, blood-related hesitancy, recalled negative experiences, and perception of the benefits associated with needle use. Distinguishing between these domains and appreciating their independence and interdependence may provide helpful insights in designing public health interventions. The ONE scale therefore represents a new parsimonious and valid instrument that may provide important actionable insights to promote global public health, and further research and testing may be warranted.

For peer review only

Domain	Item	Factor I	Factor II	Factor III	Factor IV
Injection	I am afraid of needles.	**0.9449**	0.0468	-0.0622	-0.0951
	I am afraid of being vaccinated because of the needle.	**0.9808**	-0.1095	-0.0307	0.0119
	I have experienced severe panic when thinking about or undergoing an injection.	**0.7896**	0.0227	0.1669	0.0064
	When I know I will need to be injected with a needle, I worry about it ahead of time.	**0.9088**	-0.0146	0.0406	-0.0914
	I have delayed getting a vaccine specifically because of the needle.	**0.7954**	-0.0051	0.0474	0.1659
	I am bothered by the appearance of a needle.	**0.7300**	0.2277	-0.0493	-0.0410
	I feel anxious at the thought of being injected with a needle.	**0.8713**	0.1421	-0.0257	-0.0505
	I am worried that I will feel unwell or faint if I am injected with a needle.	**0.5640**	0.2972	0.0736	-0.0564
	Needles cause significant pain.	**0.6236**	-0.1182	0.0984	0.1585
	I dislike that a needle pierces my skin and enters my body directly.	**0.7196**	-0.0200	0.0426	0.0897
Blood	I am afraid of seeing blood.	0.2112	**0.6760**	-0.0008	0.0197
	I avoid watching surgical procedures on television or on the internet.	0.1448	**0.5628**	-0.0812	-0.0174
	I feel lightheaded when I see blood.	-0.0415	**0.9135**	0.0460	0.0736
History	I had a bad experience with needles in the past.	0.0861	0.0572	**0.8677**	0.0616
	I have a strong unpleasant memory of being injected with a needle.	0.3179	-0.0119	**0.7238**	-0.0835
	I remember a nurse or doctor making a mistake when injecting me with a needle.	-0.1582	0.0021	**0.8672**	-0.0221
Benefits	I think needles are useful.	0.1967	-0.0099	-0.0248	**0.5859**
	My feelings regarding needles do not matter since needles are so useful.	-0.0694	0.0645	0.0150	**0.7389**
	People should try to overcome their fear of needles because of the benefits of using them.	-0.0999	0.0274	-0.0146	**0.8757**

Table 1. Factor loadings for the ONE EFA.

For peer review only - http://bmjopen.bmj.com/site/about/guidelines.xhtml

	Injection	Blood	History	Benefits
**Injection**	1			
**Blood**	0.59	1		
**History**	0.50	0.20	1	
**Benefits**	0.36	0.20	0.15	1

Table 2. Factor intercorrelations in the UK EFA.

For peer review only

Number	Item	Domain
1	I am afraid of needles.	Injection
2	I am afraid of being vaccinated because of the needle.	Injection
3	I have experienced severe panic when thinking about or undergoing an injection.	Injection
4	When I know I will need to be injected with a needle, I worry about it ahead of time.	Injection
5	I have delayed getting a vaccine specifically because of the needle.	Injection
6	I am bothered by the appearance of a needle.	Injection
7	I feel anxious at the thought of being injected with a needle.	Injection
8	I am worried that I will feel unwell or faint if I am injected with a needle.	Injection
9	Needles cause significant pain.	Injection
10	I dislike that a needle pierces my skin and enters my body directly.	Injection
11	I am afraid of seeing blood.	Blood
12	I avoid watching surgical procedures on television or on the internet.	Blood
13	I feel lightheaded when I see blood.	Blood
14	I had a bad experience with needles in the past.	History
15	I have a strong unpleasant memory of being injected with a needle.	History
16	I remember a nurse or doctor making a mistake when injecting me with a needle.	History
17	I think needles are useful.	Benefits
18	My feelings regarding needles do not matter since needles are so useful.	Benefits
19	People should try to overcome their fear of needles because of the benefits of using them.	Benefits

Table 3. The ONE Scale. All answer choices are rated using a 5-point Likert scale (strongly disagree though strongly agree). For questions 1-16, strongly agree is scored as 5, while for questions 17-19 strongly agree is scored as 1 (reverse scoring). Higher values reflect more hesitancy regarding needle use. The total possible ONE score ranges from 19 to 95.

For peer review only

For peer review only - http://bmjopen.bmj.com/site/about/guidelines.xhtml

	**UK Sample Median (IQR)**	**US Sample Median (IQR)**	**p-value**
Injection	17 (13-26)	19 (13-29)	0.07
Blood	7 (4-10)	7 (4-9)	0.80
History	6 (4-10)	8 (4-12)	<0.001
Benefits	5 (4-6)	5 (4-7)	0.32
ONE Scale Overall	37 (29-48)	39 (29-54)	0.054

Table 4. Median and interquartile ranges for ONE sub-scales and the overall scale. P-values are for the two-sample Wilcoxon rank-sum (Mann Whitney) test.

For peer review only

For peer review only - http://bmjopen.bmj.com/site/about/guidelines.xhtml

Subscale	UK Sample	US Sample
Injection	0.94	0.93
Blood	0.79	0.79
History	0.85	0.84
Benefits	0.71	0.70
**Overall**	**0.92**	**0.93**

Table 5. Subscale and overall reliability, as measured by McDonald's Omega.

For peer review only

References

1. World Health Organization. Ten threats to global health in 2019. 2019; https://www.who.int/news-room/spotlight/ten-threats-to-global-health-in-2019. Accessed 10/25/2022.
2. Freeman D, Loe BS, Chadwick A, et al. COVID-19 vaccine hesitancy in the UK: the Oxford coronavirus explanations, attitudes, and narratives survey (Oceans) II. *Psychological Medicine.* 2022;52(14):3127-3141.
3. Freeman D, Lambe S, Yu L-M, et al. Injection fears and COVID-19 vaccine hesitancy. *Psychological Medicine.* 2021.
4. Hettinga J, Carlisle R. Vaccination into the Dermal Compartment: Techniques, Challenges, and Prospects. *Vaccines (Basel).* 2020;8(3):534.
5. McMurtry CM, Pillai Riddell R, Taddio A, et al. Far From "Just a Poke": Common Painful Needle Procedures and the Development of Needle Fear. *The Clinical journal of pain.* 2015;31(10 Suppl):S3-11.
6. Miller MA, Pisani E. The cost of unsafe injections. *Bull World Health Organ.* 1999;77(10):808-811.
7. Mathieu E, Ritchie H, Ortiz-Ospina E, et al. A global database of COVID-19 vaccinations. *Nature Human Behaviour.* 2021;5(7):947-953.
8. Page AC, Bennett KS, Carter O, Smith J, Woodmore K. The blood-injection symptom scale (BISS): Assessing a structure of phobic symptoms elicited by blood and injections. *Behaviour Research and Therapy.* 1997;35(5):457-464.
9. van Overveld M, de Jong PJ, Peters ML. The Multi-Dimensional Blood/Injury Phobia Inventory: Its psychometric properties and relationship with disgust propensity and disgust sensitivity. *J Anxiety Disord.* 2011;25(3):319-325.
10. Olatunji BO, Ebesutani C, Sawchuk CN, McKay D, Lohr JM, Kleinknecht RA. Development and Initial Validation of the Medical Fear Survey–Short Version. *Assessment.* 2012;19(3):318-336.
11. Wenzel A, Holt CS. Validation of the Multidimensional Blood/Injury Phobia Inventory: Evidence for a unitary construct. *Journal of Psychopathology and Behavioral Assessment.* 2003;25(3):203-211.
12. Kose S, Mandiracioglu A. Fear of blood/injection in healthy and unhealthy adults admitted to a teaching hospital. *International Journal of Clinical Practice.* 2007;61(3):453-457.
13. Ovanessian MM, Fairbrother N, Vorstenbosch V, McCabe RE, Rowa K, Antony MM. Psychometric Properties and Clinical Utility of the Specific Phobia Questionnaire in an Anxiety Disorders Sample. *Journal of Psychopathology and Behavioral Assessment.* 2019;41(1):36-52.
14. Hako S, Kambara K, Ogata A. The development and validation of the multidimensional fear-of-injection scale. *Health Psychology and Behavioral Medicine.* 2022;10(1):806-817.
15. Öst L-G, Hellström K, Kåver A. One versus five sessions of exposure in the treatment of injection phobia. *Behavior Therapy.* 1992;23(2):263-281.
16. Kantor BN, Kantor J. Development and validation of the Oxford Pandemic Attitude Scale-COVID-19 (OPAS-C): an internet-based cross-sectional study in the UK and USA. *BMJ Open.* 2021;11(7):e043758-e043758.

For peer review only - http://bmjopen.bmj.com/site/about/guidelines.xhtml

17. Boateng GO, Neilands TB, Frongillo EA, Melgar-Quinonez HR, Young SL. Best Practices for Developing and Validating Scales for Health, Social, and Behavioral Research: A Primer. *Front Public Health.* 2018;6:149.
18. Watkins MW. *A step-by-step guide to exploratory factor analysis with Stata.* New York, NY: Routledge; 2022.
19. Lorenzo-Seva U, Ferrando PJ. MSA: The Forgotten Index for Identifying Inappropriate Items Before Computing Exploratory Item Factor Analysis. *Methodology.* 2021;17(4):296-306.
20. Dinno A. Implementing Horn's parallel analysis for principal component analysis and factor analysis. *Stata Journal.* 2009;9(2):291-298.
21. Horn JL. A rationale and test for the number of factors in factor analysis. *Psychometrika.* 1965;30(2):179-185.
22. Cappelleri JC, Lundy JJ, Hays RD. Overview of Classical Test Theory and Item Response Theory for the Quantitative Assessment of Items in Developing Patient-Reported Outcomes Measures Comment. *Clinical Therapeutics.* 2014;36(5):648-662.
23. Holgado–Tello FP, Chacón–Moscoso S, Barbero–García I, Vila–Abad E. Polychoric versus Pearson correlations in exploratory and confirmatory factor analysis of ordinal variables. *Quality & Quantity.* 2010;44(1):153-166.
24. Flora DB, Curran PJ. An Empirical Evaluation of Alternative Methods of Estimation for Confirmatory Factor Analysis With Ordinal Data. *Psychol Methods.* 2004;9(4):466-491.
25. Browne MW, Cudeck R. Alternative Ways of Assessing Model Fit. *Sociological Methods & Research.* 1992;21(2):230-258.
26. Browne MW, Cudeck R, Bollen KA, Long JS. *Testing structural equation models.* 1993.
27. Streiner DL. Starting at the Beginning: An Introduction to Coefficient Alpha and Internal Consistency. *Journal of Personality Assessment.* 2003;80(1):99-103.
28. Garrido LE, Abad FJ, Ponsoda V. Performance of Velicer's Minimum Average Partial Factor Retention Method With Categorical Variables. *Educational and Psychological Measurement.* 2011;71(3):551-570.
29. Ledesma RD, Valero-Mora P, Macbeth G. The Scree Test and the Number of Factors: a Dynamic Graphics Approach. *The Spanish Journal Of Psychology.*
30. Deacon B, Abramowitz J. Fear of needles and vasovagal reactions among phlebotomy patients. *Journal of Anxiety Disorders.* 2006;20(7):946-960.
31. Patakfalvi L, Benohanian A. Needle-free anaesthesia, a promising option for the needle-phobic patient. *Br J Dermatol.* 2014;170(5):1191-1192.
32. Zucoloto ML, Gonçalez T, Menezes NP, McFarland W, Custer B, Martinez EZ. Fear of blood, injections and fainting as barriers to blood donation in Brazil. *Vox Sanguinis.* 2019;114(1):pp38-46.
33. Berge KG, Agdal ML, Vika M, Skeie MS. High fear of intra-oral injections: prevalence and relationship to dental fear and dental avoidance among 10- to 16-yr-old children. *European Journal Of Oral Sciences.* 2016;124(6):pp572-579.
34. Radisic G, Duncanson E, Le Leu R, et al. Improving management of needle distress during the journey to dialysis through psychological education and training-the INJECT study feasibility pilot protocol. *Pilot Feasibility Stud.* 2022;8(1):28-28.

For peer review only

35. Olatunji BO, Sawchuk CN, Moretz MW, David B, Armstrong T, Ciesielski BG. Factor Structure and Psychometric Properties of the Injection Phobia Scale-Anxiety. *Psychological Assessment.* 2010;22(1):167-179.
36. Kleinknecht R, Kleinknecht EE, Sawchuk CN, Lee TC, Lohr J. The Medical Fear Survey: Psychometric properties. *the Behavior Therapist.* 1999;22:109-112, 119.
37. Rowan N, Wulff D. Using qualitative methods to inform scale development. *Qualitative Report.* 2007;12(3):450-466.
38. Morgado FFR, Meireles JFF, Neves CM, Amaral ACS, Ferreira MEC. Scale development: ten main limitations and recommendations to improve future research practices. *Psicol Reflex Crit.* 2017;30(1):3-20.
39. Alberts J, Löwe B, Glahn MA, et al. Development of the generic, multidimensional Treatment Expectation Questionnaire (TEX-Q) through systematic literature review, expert surveys and qualitative interviews. *BMJ Open.*
40. Boivin A, Lehoux P, Lacombe R, Burgers J, Grol RP. Involving patients in setting priorities for healthcare improvement: a cluster randomized trial. *Implement Sci.* 2014;9(1):24-24.
41. Sathanapally H, Sidhu M, Fahami R, et al. Priorities of patients with multimorbidity and of clinicians regarding treatment and health outcomes: a systematic mixed studies review. *BMJ Open.* 2020;10(2):e033445.
42. DeWees TA, Mazza GL, Golafshar MA, Dueck AC. Investigation Into the Effects of Using Normal Distribution Theory Methodology for Likert Scale Patient-Reported Outcome Data From Varying Underlying Distributions Including Floor/Ceiling Effects. *Value Health.* 2020;23(5):625-631.
43. Wu H, Leung S-O. Can Likert Scales be Treated as Interval Scales?—A Simulation Study. *Journal Of Social Service Research.* 2017;43(4):pp527-532.

For peer review only

For peer review only - http://bmjopen.bmj.com/site/about/guidelines.xhtml

Author Contributions

Study conception: JK, RCC
Study design: JK, SV, MM, AJP, RCC
Statistical analyses: JK, AJP, RCC
Writing: JK, SV, MM, AJP, RCC
Oversight: SV, MM, AJP, RCC

For peer review only

For peer review only - http://bmjopen.bmj.com/site/about/guidelines.xhtml

BMJ Open

The Oxford Needle Experience (ONE) scale: a UK- and US-based online mixed-methods psychometric development and validation study of an instrument to assess needle fear, attitudes, and expectations in the general public

Journal:	*BMJ Open*
Manuscript ID	bmjopen-2023-074466.R1
Article Type:	Original research
Date Submitted by the Author:	07-Nov-2023
Complete List of Authors:	Kantor, Jonathan; University of Oxford; University of Pennsylvania Perelman School of Medicine<br>Vanderslott, Samantha; University of Oxford<br>Morrison, Michael; University of Oxford<br>Pollard, Andrew; University of Oxford<br>Carlisle, Robert C; University of Oxford
**Primary Subject Heading**:	Public health
Secondary Subject Heading:	Epidemiology
Keywords:	Public health < INFECTIOUS DISEASES, Dialysis < NEPHROLOGY, Epidemiology < INFECTIOUS DISEASES

SCHOLARONE™
Manuscripts

I, the Submitting Author has the right to grant and does grant on behalf of all authors of the Work (as defined in the below author licence), an exclusive licence and/or a non-exclusive licence for contributions from authors who are: i) UK Crown employees; ii) where BMJ has agreed a CC-BY licence shall apply, and/or iii) in accordance with the terms applicable for US Federal Government officers or employees acting as part of their official duties; on a worldwide, perpetual, irrevocable, royalty-free basis to BMJ Publishing Group Ltd ("BMJ") its licensees and where the relevant Journal is co-owned by BMJ to the co-owners of the Journal, to publish the Work in this journal and any other BMJ products and to exploit all rights, as set out in our licence.

The Submitting Author accepts and understands that any supply made under these terms is made by BMJ to the Submitting Author unless you are acting as an employee on behalf of your employer or a postgraduate student of an affiliated institution which is paying any applicable article publishing charge ("APC") for Open Access articles. Where the Submitting Author wishes to make the Work available on an Open Access basis (and intends to pay the relevant APC), the terms of reuse of such Open Access shall be governed by a Creative Commons licence – details of these licences and which Creative Commons licence will apply to this Work are set out in our licence referred to above.

Other than as permitted in any relevant BMJ Author's Self Archiving Policies, I confirm this Work has not been accepted for publication elsewhere, is not being considered for publication elsewhere and does not duplicate material already published. I confirm all authors consent to publication of this Work and authorise the granting of this licence.

For peer review only

The Oxford Needle Experience (ONE) scale: a UK- and US-based online mixed-methods psychometric development and validation study of an instrument to assess needle fear, attitudes, and expectations in the general public

Jonathan Kantor,[1,2,3*] Samantha Vanderslott,[1] Michael Morrison,[4] Andrew J Pollard,[1] Robert C Carlisle[2]

1. Oxford Vaccine Group, University of Oxford, Oxford, UK.
2. Biomedical Ultrasonics, Biotherapy, and Biopharmaceuticals Laboratory (BUBBL), Institute of Biomedical Engineering, Department of Engineering Science, University of Oxford, Oxford, UK.
3. Center for Clinical Epidemiology and Biostatistics, University of Pennsylvania Perelman School of Medicine, Philadelphia, PA, USA.
4. Centre for Health, Law, and Emerging Technologies (HeLEX), University of Oxford, Oxford, UK.

[*]Correspondence to:

Jonathan Kantor, MD, MSc, MSCE
Oxford Vaccine Group
Biomedical Ultrasonics, Biotherapy, and Biopharmaceuticals Laboratory (BUBBL)
Institute of Biomedical Engineering
Department of Engineering Science
University of Oxford
Oxford OX3 9DU
UK
jonkantor@gmail.com
ORCID ID: 0000-0002-3256-3014

Author email addresses:

Jonathan Kantor: jonkantor@gmail.com ORCID ID: 0000-0002-3256-3014
Samantha Vanderslott: samantha.vanderslott@paediatrics.ox.ac.uk
Michael Morrison: michael.morrison@law.ox.ac.uk
Andrew J Pollard: andrew.pollard@paediatrics.ox.ac.uk
Robert C Carlisle: robert.carlisle@eng.ox.ac.uk

Abstract
Objectives: To develop and validate the Oxford Needle Experience (ONE) scale, an instrument to assess needle fear, attitudes, and expectations in the general population.
Design: Cross-sectional validation study.
Setting: Internet-based with participants in the United Kingdom (UK) and United States (US).

For peer review only

Participants: UK and US representative samples stratified by age, sex, and ethnicity using the Prolific Academic platform.
Main outcome measures: Exploratory factor analysis with categorical variables and a polychoric correlation matrix followed by promax oblique rotation on the UK sample for the ONE scale. Confirmatory factor analysis (CFA) with a Satorra-Bentler scaled test statistic evaluating the root mean squared error of approximation (RMSEA), standardized root mean squared residual (SRMR), and comparative fit index (CFI) on the US sample. Reliability as internal consistency using McDonald's omega. Convergent validity using the Pearson correlation coefficient. Predictive and discriminant validity using logistic regression odds ratios of association (OR).
Results: The population included 1,000 respondents, 500 in the UK and 500 in the US. Minimum average partial correlation and a scree plot suggested 4 factors should be retained: injection hesitancy, blood-related hesitancy, recalled negative experiences, and perceived benefits, yielding a 19-question scale. On CFA, the RMSEA was 0.070 (90% confidence interval, 0.064, 0.077), SRMR 0.053, and CFI 0.925. McDonald's omega was 0.92 and 0.93 in the UK and US samples, respectively. Convergent validity with the 4-item OCEANS needle fear scale demonstrated a strong correlation (r=0.83). Predictive validity with a single-question COVID-19 vaccination status question demonstrated a strong association, OR (95% CI) 0.97 (0.96, 0.98), p<0.0001 in the US sample. Discriminant validity with a question regarding the importance of controlling what enters the body confirmed the ONE score does not predict this unrelated outcome, OR 1.00 (0.99, 1.01), p=0.996 in the US sample.
Conclusions: The ONE scale is a reliable and valid multidimensional scale that may be useful in predicting vaccine hesitancy, designing public health interventions to improve vaccine uptake, and exploring alternatives to needles for medical procedures.

Strengths and limitations of this study

- Using an iterative qualitative methodological approach to domain development permits the inclusion of a broad and diverse stakeholder group in the development of the Oxford Needle Experience (ONE) scale.
- Relying on two large age-, sex-, and racially representative populations from both the UK and US may improve the generalisability of our findings.
- Adopting an approach that relies on polychoric correlation matrices while treating Likert-scale responses as ordinal variables may mitigate some of the statistical risks associated with treating ordinal data as continuous, thereby providing more reliable and valid results in our analysis of complex behavioural and attitudinal patterns.
- The reliance on survey panel design may introduce selection bias, as despite being demographically representative the sample may not accurately represent the general population's attitudes and experiences.
- The study focuses on the UK and US populations, potentially limiting the scale's generalisability to other cultural contexts, and additional cross-cultural validation studies may be warranted.

Keywords: needles; needle phobia; anxiety; blood-injection-injury; fear; vaccine hesitancy; vaccines; public health; validation study; behaviour rating scale; venepuncture; phlebotomy; dialysis; local anaesthetic

For peer review only

Word Count: 3,982
References: 43
Tables: 6

For peer review only

For peer review only - http://bmjopen.bmj.com/site/about/guidelines.xhtml

INTRODUCTION

Vaccine hesitancy was identified by the World Health Organization as one of the top ten threats to public health even before the onset of the COVID-19 pandemic, and the potential impact of this phenomenon on global health has only increased in recent years.(1) While vaccine hesitancy is a complex and multifactorial phenomenon, previous work has suggested that injection fear may represent its only modifiable risk factor.(2, 3) Given the importance of behavioural considerations in modulating vaccine acceptance, changes in methods of vaccine delivery may decrease vaccine hesitancy, leading to significant global health benefits.(4) With upwards of 10 billion vaccinations given by needle each year, better understanding the role of needles as potential contributors to vaccine hesitancy is of urgent public health importance, particularly as alternative approaches for vaccine delivery are undergoing development.(5-7)

Despite the central importance of understanding public attitudes to needle use for vaccination and other medical procedures, little is known regarding the myriad contributors to this phenomenon, and most previous scales designed to study needle attitudes, experiences, and fear were developed without fundamental qualitative research, in the absence of a multidimensional approach—often focusing exclusively on needle fear, anxiety, or even phobia—or in populations with suboptimal generalisability.(8-15)

Validated multidimensional scales are a useful tool to study behavioural phenomena, and may provide insight with direct global health implications.(16) Our goal, therefore, was to develop a feasible multidimensional scale that would capture the key domains contributing to concern regarding needle use in the context of vaccination or medical procedures. By better elucidating the underlying domains that may modulate vaccine hesitancy, and medical procedure hesitancy more broadly, these data may ultimately be used to better inform the development of evidence-based vaccination strategies to mitigate some of the perceived negative aspects of vaccine delivery. Moreover, a better understanding of the contribution of needles to vaccine hesitancy may also encourage the development of alternative vaccine-delivery technologies, ultimately resulting in more tailored and publicly acceptable approaches to vaccination and medical procedures in the future.

METHODS

Domain and item generation

Domain generation to establish the domains of interest for the ONE scale was accomplished through an iterative deductive and inductive approach: after a literature review was performed, online focus groups were used to qualitatively develop a set of possible themes, and ultimately domains, that putatively contribute to needle attitudes, experiences, and expectations. Online focus groups were conducted iteratively using two separate 50-respondent samples recruited through Prolific Academic. The first was a convenience sample in the UK, and the second included a group of respondents who had previously answered that they had not received any vaccinations for COVID-19. The subject was introduced by stating that 'The aim of this study is to create a survey that can be used to understand attitudes towards needles and vaccine administration via needles. The reason we are asking you to complete the survey is that in order to develop valid surveys for future use it is important for researchers to determine whether the questions that we are asking are reasonable, relatable, and understandable. It is also important for us to look at the ways in which asking similar questions in slightly ways may

For peer review only - http://bmjopen.bmj.com/site/about/guidelines.xhtml

affect responses, and how the answers to these questions group together. This will enable us to develop meaningful survey instruments in the future that can be used to better assess public attitudes.' Open ended questions included items such as 'What are five words that come to mind when you think about needles?' In addition, respondents were presented with multiple choice questions and asked to provide feedback on question quality, clarity, and saliency.

To assess the face and content validity of the scale, extensive qualitative measures were performed. The two 50-member online focus groups were asked, 'Did you feel like this set of multiple-choice questions was easy to understand? Reasonable?' to gain insights into the clarity, relevance, and interpretability of the items. This feedback mechanism was instrumental in refining the items to ensure they are aligned with the intended constructs and are easily understood. Furthermore, the iterative workshopping of items among the investigators, the Oxford Vaccine Group, the full BUBBL group at the Department of Engineering Science, and biology/ in vivo cluster meetings enriched the scale's content validity. Each item was meticulously reviewed and refined based on the collective feedback, ensuring a comprehensive and representative measure of individuals' perceptions and experiences regarding needles and injections.

A group of experts from the fields of medicine, public health, social science, and engineering science was consulted regarding domain development and inclusion, consistent with established scale development methodology.(16-18)

Participant item responses were recorded on a 5-point Likert scale ranging from strongly agree to strongly disagree. Items were reverse scored, as appropriate, to yield a scale where higher values represent greater levels of concern regarding needle use. This work was approved by the University of Oxford Medical Sciences Interdivisional Research Ethics Committee (approval reference R81585/ RE001). All subjects provided informed consent for participation and were permitted to withdraw from their anonymous surveys at any time. Qualitative analyses were performed using NVivo release 1.4 (QSR International, Burlington, Massachusetts, USA) and statistical analyses were performed using Stata for Mac version 16 (Stata Corporation, College Station, Texas, USA).

<u>Data collection</u>

Two separate representative samples were used in the development of the ONE. A sample of 500 respondents in the United Kingdom (UK) was recruited through Prolific Academic (Oxford, UK), an academic survey panel site, and a draft scale was presented using the Qualtrics platform (Qualtrics Corp, Provo, Utah, USA). All respondents were rewarded with a small payment (<£2). This sample was used to develop the scale and perform exploratory factor analysis (EFA). A second demographically representative sample of respondents from the United States (US) was then solicited in a similar fashion using the same electronic survey recruitment tools. This sample was used for confirmatory factor analysis and reliability and validity testing. Results from the US and UK were evaluated separately to establish the robustness of the scale. Demographic information was self-reported by respondents and was linked with responses through an anonymous 25-digit alphanumeric key. Baseline information included age, self-reported sex, self-reported race and ethnicity, employment status, education history, household income, and vaccination status against COVID-19.

For peer review only - http://bmjopen.bmj.com/site/about/guidelines.xhtml

Domain and item reduction and exploratory factor analysis

The Kaiser-Meyer-Olkin procedure was conducted to ensure sampling adequacy.(19) To establish the number of domains to retain for the ONE scale, we calculated the minimum average partial correlation for the number of principal components, developed a scree plot, and performed parallel analysis for principal components.(18, 20, 21) Item reduction was approached by assessing domain relevance, examining polychoric (item-item) and polyserial (item-test) correlations, culling redundant items, and removing those with low communality or low factor loadings.(22) EFA with categorical variables was performed by using polychoric correlations to generate a factor matrix using iterated principal factor analysis followed by promax oblique factor rotation.(23, 24)

Confirmatory factor analysis

The robustness of the findings was tested by performing confirmatory maximum likelihood factor analysis using structural equation modelling on the US sample with the model developed in the UK sample with a Satorra-Bentler scaled test statistic. Multiple goodness of fit indices were analysed, including the root mean squared error of approximation (RMSEA), the standardized root mean squared residual (SRMR), and the comparative fit index (CFI) to assess model fit. Values of RMSEA≤0.08, SRMR≤0.08, and CFI≥0.90 suggest acceptable fit.(25, 26)

Reliability and validity

Reliability as internal consistency was assessed by examining McDonald's omega, a more robust alternative to Cronbach's alpha, for each subscale domain and for the overall scale, in both the UK and US samples.(27)

Convergent validity was evaluated by examining the correlation between the ONE scale and the 4-item OCEAN fears factor score.[3] Predictive validity was assessed by examining the association between the ONE scale score and vaccination status against COVID-19. Logistic regression odds ratios of association were calculated with vaccination status (defined as having received a minimum of one dose of any vaccine for COVID-19) coded as a dichotomous variable and the ONE scale treated as a continuous predictor variable. Discriminant validity was assessed by examining the logistic regression odds ratios of association between the ONE score and agreement with a non-specific statement ('it is important to control what enters one's body') in the UK and US samples. A p-value of 0.05 was considered significant.

Patient and public involvement

This study was conducted to develop a scale to assess public attitudes to needle use for medical procedures such as vaccination. Patient and public involvement was not actively solicited for the initial phase of study design, and patients and members of the public were primarily participating as survey respondents. However, patients and members of the public taking part in online focus groups on needle hesitancy were able to provide feedback on the aims of the project as well as the suitability of the wording of specific scale items. The aim of this research is to develop a reliable scale that reflects the priorities and preferences of the public. Results will be disseminated and may potentially be used in future studies to better assess patient and public preferences regarding the use of needles in vaccination and in medical procedure more broadly.

For peer review only - http://bmjopen.bmj.com/site/about/guidelines.xhtml

RESULTS

Respondent characteristics

In the UK sample, of the 506 respondents who started the survey, 500 subjects returned completed surveys, yielding a 98.8% completion rate in the survey panel. The median (interquartile range [IQR]) age was 45 (32, 59), and respondents ranged in age from 18 to 90. 50.4% of respondents identified as female, 48.2% as male, and 1% as non-binary. The median (IQR) self-reported household income was £39,000 (25,000, 58,000). A total of 47.6% of respondents were currently working full-time and 14.2% identified as non-white.

In the US sample, of the 509 respondents who started the survey, 500 subjects returned completed surveys, yielding a 98.2% completion rate in the survey panel. The median (IQR) age was 45 (31, 60), and respondents ranged in age from 18 to 84. 49.6% of respondents identified as female, 47.6% as male, and 1.6% as non-binary. The median (IQR) self-reported household income was $53,500 (31,000, 89,999). A total of 45.5% of respondents were currently working full-time and 27.8% identified as non-white.

Domain and item reduction and exploratory factor analysis

A total of 16 themes were identified as potentially contributing to public attitudes to needles after literature review and qualitative analysis of online focus group data. The themes identified through a literature review included fear, history of negative experiences, intrusive thoughts, risk, avoidance, needle appearance, fainting, anxiety, pain, blood, and trust. The focus group feedback added several additional themes including general dislike, concerns regarding personal control, fears regarding maintaining bodily integrity, positive aspects of needle use, and the training/expertise/skill of the injector. A total of 107 items for potential scale inclusion were then developed for testing; the intentionally large domain and item pool was designed to pilot similar iterations of items in a focus group format. A total of 88 items were ultimately deleted due to low domain relevance, low polychoric or polyserial correlations, unifactorial outcomes, redundancy, or low communality.

Factor loadings from the 19 retained items after EFA for categorical variables with promax oblique rotation are presented in Table 1. The Kaiser-Meyer-Olkin measure of sampling adequacy was excellent, with a value of 0.92 for the overall model and with values ranging from 0.68 to 0.97 for each of the individual items. This 4-factor solution reflected a simple structure with appropriate loading and was supported by the results of the minimum average partial correlation analysis, the scree plot, and the Eigenvalue > 1 approaches to factor extraction.(28, 29) Parallel analysis suggested retaining 3 factors, though this result was considered borderline given the adjusted Eigenvalue of 0.97 for the fourth factor, just below the cut-off of 1.(20)

Factors correlated clinically with the domains of 1) injection hesitancy (including fear and anxiety related to injection); 2) blood-related hesitancy; 3) recalled negative experiences; and 4) perceived benefits of needles.

Factor intercorrelations are included in Table 2, and the final ONE scale and scoring key are presented in Table 3.

Confirmatory factor analysis

For peer review only - http://bmjopen.bmj.com/site/about/guidelines.xhtml

Confirmatory factor analysis was performed on the US sample using the model developed in the UK sample. The maximum likelihood factor analysis model created using structural equation modelling demonstrated acceptable goodness of fit characteristics, with RMSEA=0.070 (90% confidence interval 0.064, 0.077), SRMR=0.053, and CFI=0.925 using the 4-factor model with a total of 19 items. Standardized factor loadings are presented in the Supplemental Table.

The median (interquartile range [IQR]) total ONE score was 37 (29-48) in the UK population and 39 (29-54) in the US population (p=0.054). Further descriptive statistics for each subscale are included in Table 4.

Reliability and validity

Reliability as internal consistency was excellent: McDonald's omega for the overall ONE scale was 0.92 in the UK sample and 0.93 in the US sample. Omega values for each subscale are included in Table 5.

Convergent validity with the OCEAN needle fear score supported our validity argument, showing a strong correlation between the scales (r=0.83). Predictive validity with the single-question vaccination status question demonstrated a significant association between vaccination status and ONE score, with a logistic regression odds ratio of association (95% CI) of 0.98 (0.96, 1.0), p=0.014 for the UK sample and 0.97 (0.96, 0.98), p<0.0001 for the US population. Given that the standard deviation of the ONE score in the UK and US samples is 15.2 and 16.5, respectively, this translates into a decrease in the odds of being vaccinated of 30.4% in the UK and 49.5% in the US for each single standard deviation increase in ONE score.

Discriminant validity with a question regarding the importance of controlling what enters the body confirmed that the ONE score does not predict this unrelated outcome, with logistic regression odds ratios of association of 1.01 (1.00, 1.02), p=0.162 in the UK population and 1.00 (0.99, 1.01), p=0.996 in the US sample.

DISCUSSION

We have shown here that the ONE scale is a reliable multidimensional instrument and that there is significant preliminary evidence to support the scale's validity for assessing public attitudes to needle use in the context of medical procedures such as vaccination. Given that injection fears may be the primary modifiable risk factor for vaccine refusal,(3) understanding the contributors to such responses is of vital public health importance. Moreover, needle concerns are not limited to those undergoing vaccination, and have been implicated in hesitancy regarding a range of procedures from venepuncture(30) to local anaesthetic injection in dermatologic surgery,(31) blood donation,(32) dental procedures,(33) and dialysis.(34) Therefore, a better appreciation of the myriad contributors to the needle exposure experience may be broadly valuable.

In evaluating descriptive statistics for each of the subscales, only the median scores for a recalled history of negative experiences differed significantly between the UK and US populations, with an absolute 33% increase reported in the US group (p<0.0001). As such recall has been previously implicated as a potential driver of needle fear—where a feedback loop of recalled negative experiences leads to avoidance, furthering fear—this may have important implications for needle acceptance.(5) Moreover, the magnitude of the association between ONE score and COVID-19 vaccination status—the odds of being unvaccinated increase by

For peer review only - http://bmjopen.bmj.com/site/about/guidelines.xhtml

almost 50% for each standard deviation increase in ONE score in the US population—not only highlights the predictive validity of the scale but also underscores its utility and potential value in designing public health interventions and the potential role of needle hesitancy as a contributor to vaccine hesitancy.(3)

Several prior instruments have been developed to assess needle attitudes. The 18-item Injection Phobia Scale-Anxiety (IPS-Anx) was developed in 1992 on a sample of 40 subjects with known needle phobia and examines fear and anxiety exclusively.(15) Two decades after its initial development, the factorial structure of the scale was assessed, suggesting a two-factor structure, encompassing contact (direct injection) and distal (watching or discussing injection) fears.(35) The 50-item Medical Fear Survey examines an array of fears relating to medical procedures, including injections as well as blood, physical examination, and mutilation;(36) an abbreviated version of this survey has also been developed.(10) The blood/injection fear scale was developed on a Turkish population of patients attending a hospital for examination or blood donation and is a 20-question scale that includes two domains, injection and blood.(12) The Blood-Injection Symptom Scale assesses domains of faintness, anxiety, and tension, and relied on a population of Australian university students for its development.(8) The 40-item Multidimensional Blood/ Injury Phobia Inventory (MBPI) was developed on a US university student population to assess phobias, and is aimed at assessing pathological reactions, rather than understanding the range of experiences of the general population exposed to needles.(9, 11) Blood-injection-injury (BII) was also assessed as part of a 10-item subscale of the Specific Phobia Questionnaire, a lengthy scale addressing phobias ranging from fear of animals to the natural environment; this scale was developed on a cohort of patients with known anxiety disorders and a group of Canadian university students.(13) Finally, the multidimensional fear of injection scale was developed on 419 Japanese university students to evaluate fear of injections exclusively, encompassing domains of direct fear, indirect fear, physiological response, and avoidance.(14)

Unlike these scales, we used a qualitative methodology to ensure that our domain- and item-generation would include the diversity of priorities of a wide range of respondents to the experience of undergoing injection with a needle—a methodology designed to capture the broadest possible themes that may not be obvious to investigators a priori.(17, 37-39) This is important, as prior studies have suggested a disconnect between clinicians' perception of patient or public priorities and their actual priorities.(40, 41) The ONE scale is also the only instrument to include domains that assess recalled negative experiences as well as positive beliefs regarding the utility of needles, which may add significantly to researchers' understanding of patient and public priorities.

Additional strengths of our study include the iterative approach and the use of representative populations in our exploratory and confirmatory factor analysis; many prior studies relied on less diverse populations of young, well-educated students—or, conversely, on those being treated for anxiety disorders—and therefore the generalisability of their scales to the wider population is unknown. Furthermore, some prior scales aimed to evaluate pathological responses to needle injection—needle phobia. While such work is highly valuable, our goal was to develop a scale that could be broadly adopted for designing public health interventions and alternative vaccine and medical therapeutic delivery technologies for the public.

For peer review only - http://bmjopen.bmj.com/site/about/guidelines.xhtml

We also relied on statistical analyses appropriate for ordinal data, including a polychoric-matrix based EFA, to provide conservative estimates given concerns regarding the assumed normality of Likert-scale data.(42, 43) This represents a departure from earlier studies that acknowledged the skewness inherent in needle fear assessments—the majority of individuals have only mild symptoms while a small proportion have extreme reactions—but used statistics more appropriate for analysing normally-distributed data with continuous endpoints.(11)

The samples from the UK and US were designed to be demographically representative. Prolific Academic creates representative samples in the UK and US populations by stratifying by age (across five 9-year age brackets), sex (male or female), and ethnicity (using the five simplified categories recommended by the UK Office of National Statistics: White, Mixed, Asian, Black and Other). Cross stratifying by age (5 brackets), sex (2 groups), and ethnicity (5 groups) yields 50 subgroups. The number of respondents in each subgroup is designed to match census data from the US Census Bureau (2015) or the UK Office of National Statistics (2011), as appropriate, and allocations to each subgroup are made proportionately. Subjects are recruited from the survey panel site, whose current database includes over 130,000 identity-confirmed participants, and subjects are offered to participate if the particular subgroup to which they belong (for example, an Asian male between 18 and 27) has not yet been filled. We acknowledge that a panel-based sampling method has biases, though the large sample size and broad range of respondent characteristics suggests that this approach yields insights that are broadly applicable to the general populations of the UK and US.

Our work has several important limitations. First, we relied on a survey panel design to allow for a demographically representative population from both the UK and US, but there may be important differences between the general population and the subset willing to volunteer to contribute to survey panels, and response rates cannot be calculated with this approach. Moreover, all research that relies on self-report introduces the risk that bias, and particularly response bias and social desirability bias, may affect stated preferences. Nevertheless, the robustness of our findings, with total and subscale scores largely similar across both the UK and US populations, supports the generalisability of the scale. Second, one domain—injection hesitancy—accounts for 10 of the 19 items in the scale, while the remaining domains each include 3 items. The unequal number of items across domains, however, was purposely designed to capture the presence of a primary domain of interest—injection hesitancy—while also acknowledging and evaluating the contribution of other domains. Indeed, some have suggested that a unitary construct underlies responses to needle injection,(11) and it was our goal to expand on this central domain rather than attempt to over-extract factors that otherwise correlate closely. Third, additional validation testing, as well as test-retest reliability and cross-cultural validation studies, should be considered in the future to better understand the limits of the ONE scale's generalisability, particularly given the potential for cultural and spiritual beliefs to affect these results. Fourth, an additional limitation of our manuscript is the absence of invariance testing and multi-group comparisons. We did not assess whether the ONE scale's item scores are comparable between subgroups based on demographic variables, such as country of origin, age, and socioeconomic status, or determine whether the scale operates equivalently across different groups regarding the perceived item content and the dimensionality of underlying constructs. Future research including such analyses may aid in

For peer review only - http://bmjopen.bmj.com/site/about/guidelines.xhtml

establishing the scale's robustness across diverse clinical and research settings. Finally, an added limitation of our methodological approach was the lack of formal cognitive interviews performed to establish face and content validity. While we acknowledge that our approach does not provide an absolute guarantee of item interpretation consistency, the thorough and iterative review and feedback process bolsters the face and content validity of the ONE scale. Though the focus group data and feedback from experts was instrumental in developing the scale, future studies including cognitive interviews may be helpful to further support arguments for the validity of the ONE scale for assessing needle attitudes.

The ONE scale addresses four separate domains that contribute to the public's experience with needles: injection hesitancy, blood-related hesitancy, recalled negative experiences, and perception of the benefits associated with needle use. Distinguishing between these domains and appreciating their independence and interdependence may provide helpful insights in designing public health interventions. Indeed, as with any multidimensional scale, when interpreting the total ONE score it is important to appreciate that different domains (subscales) contribute in varying amounts to the scoring and may offset each other. Injection hesitancy accounts for the lion's share of the variance, and includes 10 of the 19 total score item, but this can be offset by scores on the other domains. While the correlations between the injection subscale and blood-related hesitancy subscale is fairly strong (0.59), some of the other subscales correlate poorly—for example, the correlation between the perceived benefits of needle use and other subscales ranges from 0.15 to 0.36. On a practical level, this means that while subscale data add important granular detail, they may also substantially offset each other, potentially diluting the actionable utility of the global score in understanding contributors to needle attitudes. Therefore, as with any multidimensional scale, the overall score provides useful summary information but for deeper insights attention must be directed at the subscale scoring level. The ONE scale represents a new parsimonious instrument that may provide important actionable insights to promote global public health, and further research and testing may be warranted.

Contributors

Study conception: JK, RCC. Study design: JK, SV, MM, AJP, RCC. Statistical analyses: JK, AJP, RCC. Writing: JK, SV, MM, AJP, RCC. Oversight: SV, MM, AJP, RCC.

Funding

SV received funding from AHRC-IRC (HNR02360) and NIHR (HNR02910). RCC is grateful for the generous benefaction of Mr Donald Porteus which supported his contribution. The funding agencies and parties were not involved in study design, data collection, analysis, or presentation.

Competing interests

The authors have no competing interests to declare.

Data availability statement

For peer review only - http://bmjopen.bmj.com/site/about/guidelines.xhtml

Data are available from the corresponding author on reasonable request.

For peer review only

For peer review only - http://bmjopen.bmj.com/site/about/guidelines.xhtml

Domain	Item	Factor I	Factor II	Factor III	Factor IV
Injection	I am afraid of needles.	**0.9449**	0.0468	-0.0622	-0.0951
	I am afraid of being vaccinated because of the needle.	**0.9808**	-0.1095	-0.0307	0.0119
	I have experienced severe panic when thinking about or undergoing an injection.	**0.7896**	0.0227	0.1669	0.0064
	When I know I will need to be injected with a needle, I worry about it ahead of time.	**0.9088**	-0.0146	0.0406	-0.0914
	I have delayed getting a vaccine specifically because of the needle.	**0.7954**	-0.0051	0.0474	0.1659
	I am bothered by the appearance of a needle.	**0.7300**	0.2277	-0.0493	-0.0410
	I feel anxious at the thought of being injected with a needle.	**0.8713**	0.1421	-0.0257	-0.0505
	I am worried that I will feel unwell or faint if I am injected with a needle.	**0.5640**	0.2972	0.0736	-0.0564
	Needles cause significant pain.	**0.6236**	-0.1182	0.0984	0.1585
	I dislike that a needle pierces my skin and enters my body directly.	**0.7196**	-0.0200	0.0426	0.0897
Blood	I am afraid of seeing blood.	0.2112	**0.6760**	-0.0008	0.0197
	I avoid watching surgical procedures on television or on the internet.	0.1448	**0.5628**	-0.0812	-0.0174
	I feel lightheaded when I see blood.	-0.0415	**0.9135**	0.0460	0.0736
History	I had a bad experience with needles in the past.	0.0861	0.0572	**0.8677**	0.0616
	I have a strong unpleasant memory of being injected with a needle.	0.3179	-0.0119	**0.7238**	-0.0835
	I remember a nurse or doctor making a mistake when injecting me with a needle.	-0.1582	0.0021	**0.8672**	-0.0221
Benefits	I think needles are useful.	0.1967	-0.0099	-0.0248	**0.5859**
	My feelings regarding needles do not matter since needles are so useful.	-0.0694	0.0645	0.0150	**0.7389**
	People should try to overcome their fear of needles because of the benefits of using them.	-0.0999	0.0274	-0.0146	**0.8757**

Table 1. Factor loadings for the ONE EFA

For peer review only

	Injection	Blood	History	Benefits
**Injection**	1			
**Blood**	0.59	1		
**History**	0.50	0.20	1	
**Benefits**	0.36	0.20	0.15	1

Table 2. Factor intercorrelations in the UK EFA

For peer review only

Number	Item	Domain
1	I am afraid of needles.	Injection
2	I am afraid of being vaccinated because of the needle.	Injection
3	I have experienced severe panic when thinking about or undergoing an injection.	Injection
4	When I know I will need to be injected with a needle, I worry about it ahead of time.	Injection
5	I have delayed getting a vaccine specifically because of the needle.	Injection
6	I am bothered by the appearance of a needle.	Injection
7	I feel anxious at the thought of being injected with a needle.	Injection
8	I am worried that I will feel unwell or faint if I am injected with a needle.	Injection
9	Needles cause significant pain.	Injection
10	I dislike that a needle pierces my skin and enters my body directly.	Injection
11	I am afraid of seeing blood.	Blood
12	I avoid watching surgical procedures on television or on the internet.	Blood
13	I feel lightheaded when I see blood.	Blood
14	I had a bad experience with needles in the past.	History
15	I have a strong unpleasant memory of being injected with a needle.	History
16	I remember a nurse or doctor making a mistake when injecting me with a needle.	History
17	I think needles are useful.	Benefits
18	My feelings regarding needles do not matter since needles are so useful.	Benefits
19	People should try to overcome their fear of needles because of the benefits of using them.	Benefits

Table 3. The ONE Scale

All answer choices are rated using a 5-point Likert scale (strongly disagree though strongly agree). For questions 1-16, strongly agree is scored as 5, while for questions 17-19 strongly agree is scored as 1 (reverse scoring). Higher values reflect more hesitancy regarding needle use. The total possible ONE score ranges from 19 to 95.

For peer review only

For peer review only - http://bmjopen.bmj.com/site/about/guidelines.xhtml

	UK Sample Median (IQR)	US Sample Median (IQR)	p-value*
Injection	17 (13-26)	19 (13-29)	0.07
Blood	7 (4-10)	7 (4-9)	0.80
History	6 (4-10)	8 (4-12)	<0.001
Benefits	5 (4-6)	5 (4-7)	0.32
ONE Scale Overall	37 (29-48)	39 (29-54)	0.054

Table 4. Median and interquartile ranges for ONE sub-scales and the overall scale
*p-values are for the two-sample Wilcoxon rank-sum (Mann Whitney) test.

For peer review only

Subscale	UK Sample	US Sample
Injection	0.94	0.93
Blood	0.79	0.79
History	0.85	0.84
Benefits	0.71	0.70
**Overall**	**0.92**	**0.93**

Table 5. Subscale and overall reliability, as measured by McDonald's Omega

For peer review only

References

1. World Health Organization. Ten threats to global health in 2019. 2019; https://www.who.int/news-room/spotlight/ten-threats-to-global-health-in-2019. Accessed 10/25/2022.
2. Freeman D, Loe BS, Chadwick A, et al. COVID-19 vaccine hesitancy in the UK: the Oxford coronavirus explanations, attitudes, and narratives survey (Oceans) II. *Psychological Medicine.* 2022;52(14):3127-3141.
3. Freeman D, Lambe S, Yu L-M, et al. Injection fears and COVID-19 vaccine hesitancy. *Psychological Medicine.* 2021.
4. Hettinga J, Carlisle R. Vaccination into the Dermal Compartment: Techniques, Challenges, and Prospects. *Vaccines (Basel).* 2020;8(3):534.
5. McMurtry CM, Pillai Riddell R, Taddio A, et al. Far From "Just a Poke": Common Painful Needle Procedures and the Development of Needle Fear. *The Clinical journal of pain.* 2015;31(10 Suppl):S3-11.
6. Miller MA, Pisani E. The cost of unsafe injections. *Bull World Health Organ.* 1999;77(10):808-811.
7. Mathieu E, Ritchie H, Ortiz-Ospina E, et al. A global database of COVID-19 vaccinations. *Nature Human Behaviour.* 2021;5(7):947-953.
8. Page AC, Bennett KS, Carter O, Smith J, Woodmore K. The blood-injection symptom scale (BISS): Assessing a structure of phobic symptoms elicited by blood and injections. *Behaviour Research and Therapy.* 1997;35(5):457-464.
9. van Overveld M, de Jong PJ, Peters ML. The Multi-Dimensional Blood/Injury Phobia Inventory: Its psychometric properties and relationship with disgust propensity and disgust sensitivity. *J Anxiety Disord.* 2011;25(3):319-325.
10. Olatunji BO, Ebesutani C, Sawchuk CN, McKay D, Lohr JM, Kleinknecht RA. Development and Initial Validation of the Medical Fear Survey–Short Version. *Assessment.* 2012;19(3):318-336.
11. Wenzel A, Holt CS. Validation of the Multidimensional Blood/Injury Phobia Inventory: Evidence for a unitary construct. *Journal of Psychopathology and Behavioral Assessment.* 2003;25(3):203-211.
12. Kose S, Mandiracioglu A. Fear of blood/injection in healthy and unhealthy adults admitted to a teaching hospital. *International Journal of Clinical Practice.* 2007;61(3):453-457.
13. Ovanessian MM, Fairbrother N, Vorstenbosch V, McCabe RE, Rowa K, Antony MM. Psychometric Properties and Clinical Utility of the Specific Phobia Questionnaire in an Anxiety Disorders Sample. *Journal of Psychopathology and Behavioral Assessment.* 2019;41(1):36-52.
14. Hako S, Kambara K, Ogata A. The development and validation of the multidimensional fear-of-injection scale. *Health Psychology and Behavioral Medicine.* 2022;10(1):806-817.
15. Öst L-G, Hellström K, Kåver A. One versus five sessions of exposure in the treatment of injection phobia. *Behavior Therapy.* 1992;23(2):263-281.
16. Kantor BN, Kantor J. Development and validation of the Oxford Pandemic Attitude Scale-COVID-19 (OPAS-C): an internet-based cross-sectional study in the UK and USA. *BMJ Open.* 2021;11(7):e043758-e043758.

For peer review only - http://bmjopen.bmj.com/site/about/guidelines.xhtml

17. Boateng GO, Neilands TB, Frongillo EA, Melgar-Quinonez HR, Young SL. Best Practices for Developing and Validating Scales for Health, Social, and Behavioral Research: A Primer. *Front Public Health.* 2018;6:149.
18. Watkins MW. *A step-by-step guide to exploratory factor analysis with Stata.* New York, NY: Routledge; 2022.
19. Lorenzo-Seva U, Ferrando PJ. MSA: The Forgotten Index for Identifying Inappropriate Items Before Computing Exploratory Item Factor Analysis. *Methodology.* 2021;17(4):296-306.
20. Dinno A. Implementing Horn's parallel analysis for principal component analysis and factor analysis. *Stata Journal.* 2009;9(2):291-298.
21. Horn JL. A rationale and test for the number of factors in factor analysis. *Psychometrika.* 1965;30(2):179-185.
22. Cappelleri JC, Lundy JJ, Hays RD. Overview of Classical Test Theory and Item Response Theory for the Quantitative Assessment of Items in Developing Patient-Reported Outcomes Measures Comment. *Clinical Therapeutics.* 2014;36(5):648-662.
23. Holgado–Tello FP, Chacón–Moscoso S, Barbero–García I, Vila–Abad E. Polychoric versus Pearson correlations in exploratory and confirmatory factor analysis of ordinal variables. *Quality & Quantity.* 2010;44(1):153-166.
24. Flora DB, Curran PJ. An Empirical Evaluation of Alternative Methods of Estimation for Confirmatory Factor Analysis With Ordinal Data. *Psychol Methods.* 2004;9(4):466-491.
25. Browne MW, Cudeck R. Alternative Ways of Assessing Model Fit. *Sociological Methods & Research.* 1992;21(2):230-258.
26. Browne MW, Cudeck R, Bollen KA, Long JS. *Testing structural equation models.* 1993.
27. Streiner DL. Starting at the Beginning: An Introduction to Coefficient Alpha and Internal Consistency. *Journal of Personality Assessment.* 2003;80(1):99-103.
28. Garrido LE, Abad FJ, Ponsoda V. Performance of Velicer's Minimum Average Partial Factor Retention Method With Categorical Variables. *Educational and Psychological Measurement.* 2011;71(3):551-570.
29. Ledesma RD, Valero-Mora P, Macbeth G. The Scree Test and the Number of Factors: a Dynamic Graphics Approach. *The Spanish Journal Of Psychology.*
30. Deacon B, Abramowitz J. Fear of needles and vasovagal reactions among phlebotomy patients. *Journal of Anxiety Disorders.* 2006;20(7):946-960.
31. Patakfalvi L, Benohanian A. Needle-free anaesthesia, a promising option for the needle-phobic patient. *Br J Dermatol.* 2014;170(5):1191-1192.
32. Zucoloto ML, Gonçalez T, Menezes NP, McFarland W, Custer B, Martinez EZ. Fear of blood, injections and fainting as barriers to blood donation in Brazil. *Vox Sanguinis.* 2019;114(1):pp38-46.
33. Berge KG, Agdal ML, Vika M, Skeie MS. High fear of intra-oral injections: prevalence and relationship to dental fear and dental avoidance among 10- to 16-yr-old children. *European Journal Of Oral Sciences.* 2016;124(6):pp572-579.
34. Radisic G, Duncanson E, Le Leu R, et al. Improving management of needle distress during the journey to dialysis through psychological education and training-the INJECT study feasibility pilot protocol. *Pilot Feasibility Stud.* 2022;8(1):28-28.

For peer review only - http://bmjopen.bmj.com/site/about/guidelines.xhtml

35. Olatunji BO, Sawchuk CN, Moretz MW, David B, Armstrong T, Ciesielski BG. Factor Structure and Psychometric Properties of the Injection Phobia Scale-Anxiety. *Psychological Assessment.* 2010;22(1):167-179.
36. Kleinknecht R, Kleinknecht EE, Sawchuk CN, Lee TC, Lohr J. The Medical Fear Survey: Psychometric properties. *the Behavior Therapist.* 1999;22:109-112, 119.
37. Rowan N, Wulff D. Using qualitative methods to inform scale development. *Qualitative Report.* 2007;12(3):450-466.
38. Morgado FFR, Meireles JFF, Neves CM, Amaral ACS, Ferreira MEC. Scale development: ten main limitations and recommendations to improve future research practices. *Psicol Reflex Crit.* 2017;30(1):3-20.
39. Alberts J, Löwe B, Glahn MA, et al. Development of the generic, multidimensional Treatment Expectation Questionnaire (TEX-Q) through systematic literature review, expert surveys and qualitative interviews. *BMJ Open.*
40. Boivin A, Lehoux P, Lacombe R, Burgers J, Grol RP. Involving patients in setting priorities for healthcare improvement: a cluster randomized trial. *Implement Sci.* 2014;9(1):24-24.
41. Sathanapally H, Sidhu M, Fahami R, et al. Priorities of patients with multimorbidity and of clinicians regarding treatment and health outcomes: a systematic mixed studies review. *BMJ Open.* 2020;10(2):e033445.
42. DeWees TA, Mazza GL, Golafshar MA, Dueck AC. Investigation Into the Effects of Using Normal Distribution Theory Methodology for Likert Scale Patient-Reported Outcome Data From Varying Underlying Distributions Including Floor/Ceiling Effects. *Value Health.* 2020;23(5):625-631.
43. Wu H, Leung S-O. Can Likert Scales be Treated as Interval Scales?—A Simulation Study. *Journal Of Social Service Research.* 2017;43(4):pp527-532.

For peer review only

For peer review only - http://bmjopen.bmj.com/site/about/guidelines.xhtml

Latent Variable	Item	Standardized Factor Loadings	Std. Err.	z-value	P-value
Injection	1	1 (constrained)	-	-	-
Injection	2	0.897	0.043	20.77	<0.0001
Injection	3	0.878	0.044	19.92	<0.0001
Injection	4	1.149	0.042	27.43	<0.0001
Injection	5	0.673	0.052	12.99	<0.0001
Injection	6	0.997	0.042	23.78	<0.0001
Injection	7	0.859	0.052	16.50	<0.0001
Injection	8	1.180	0.043	27.52	<0.0001
Injection	9	0.658	0.048	13.68	<0.0001
Injection	10	0.955	0.048	19.73	<0.0001
Blood	11	1 (constrained)	-	-	-
Blood	12	0.928	0.063	14.80	<0.0001
Blood	13	1.008	0.057	17.58	<0.0001
History	14	1 (constrained)	-	-	-
History	15	1.135	0.044	25.89	<0.0001
History	16	0.837	0.051	16.36	<0.0001
Benefits	17	1 (constrained)	-	-	-
Benefits	18	1.860	0.243	7.66	<0.0001
Benefits	19	1.850	0.236	7.83	<0.0001

Supplemental Table. Standardized factor loadings of the ONE scale using structural equation modeling.

For peer review only
